# TMEM43 Mutation p.S358L Alters Intercalated Disc Protein Expression and Reduces Conduction Velocity in Arrhythmogenic Right Ventricular Cardiomyopathy

**DOI:** 10.1371/journal.pone.0109128

**Published:** 2014-10-24

**Authors:** Vinayakumar Siragam, Xuezhi Cui, Stephane Masse, Cameron Ackerley, Shabana Aafaqi, Linn Strandberg, Michael Tropak, Michael D. Fridman, Kumaraswamy Nanthakumar, Jun Liu, Yu Sun, Bin Su, Caroline Wang, Xiaoru Liu, Yuqing Yan, Ariel Mendlowitz, Robert M. Hamilton

**Affiliations:** 1 Physiology and Experimental Medicine, The Hospital for Sick Children and Research Institute, Toronto, Ontario, Canada; 2 Division of Cardiology, University Health Network, Toronto, Ontario, Canada; 3 Division of Pathology, The Hospital for Sick Children and Research Institute, Toronto, Ontario, Canada; 4 Genetics and Genome Biology, The Hospital for Sick Children and Research Institute, Toronto, Ontario, Canada; 5 Advanced Micro and Nanosystems Laboratory, Department of Mechanical and Industrial Engineering, University of Toronto, Toronto, Ontario, Canada; Loyola University Chicago, United States of America

## Abstract

Arrhythmogenic right ventricular cardiomyopathy (ARVC) is a myocardial disease characterized by fibro-fatty replacement of myocardium in the right ventricular free wall and frequently results in life-threatening ventricular arrhythmias and sudden cardiac death. A heterozygous missense mutation in the transmembrane protein 43 (TMEM43) gene, p.S358L, has been genetically identified to cause autosomal dominant ARVC type 5 in a founder population from the island of Newfoundland, Canada. Little is known about the function of the TMEM43 protein or how it leads to the pathogenesis of ARVC. We sought to determine the distribution of TMEM43 and the effect of the p.S358L mutation on the expression and distribution of various intercalated (IC) disc proteins as well as functional effects on IC disc gap junction dye transfer and conduction velocity in cell culture. Through Western blot analysis, transmission electron microscopy (TEM), immunofluorescence (IF), and electrophysiological analysis, our results showed that the stable expression of p.S358L mutation in the HL-1 cardiac cell line resulted in decreased Zonula Occludens (ZO-1) expression and the loss of ZO-1 localization to cell-cell junctions. Junctional Plakoglobin (JUP) and α-catenin proteins were redistributed to the cytoplasm with decreased localization to cell-cell junctions. Connexin-43 (Cx43) phosphorylation was altered, and there was reduced gap junction dye transfer and conduction velocity in mutant TMEM43-transfected cells. These observations suggest that expression of the p.S358L mutant of TMEM43 found in ARVC type 5 may affect localization of proteins involved in conduction, alter gap junction function and reduce conduction velocity in cardiac tissue.

## Introduction

TMEM43 (also called LUMA) [1] is a 43 kDa putative membrane protein of undetermined structure and function. A heterozygous TMEM43 gene mutation causes the type 5 autosomal dominant form of arrhythmogenic right ventricular cardiomyopathy (ARVC) identified in a founder population on the island province of Newfoundland in Canada [2], but is being increasingly identified in other populations, and may have been imported from continental Europe. [3]–[5]. ARVC is a heritable cardiomyopathy that is being increasingly recognized as a major cause of sudden cardiac death [6] [7], [8] and has been associated with up to 20% of sudden deaths among young people [9]. Sudden cardiac death in ARVC is believed to result from re-entrant ventricular arrhythmias due to a combination of factors including mechanical failure of intercalated (IC) discs, fibro-fatty infiltration of the myocardium and reduced connexin-43 (Cx43) gap junction conduction between cells in the myocardial syncytium.

The TMEM43 heterozygous missense mutation implicated in ARVC type 5 (ARVC5) in Newfoundland (c.1073C>T; p.S358L) was found in 15 Newfoundland families with a common a disease-associated haplotype [2]. This gene mutation was identified through fine mapping of the ARVC5 locus at 3p23 followed by sequencing of positional candidate genes. It was shared by all clinically affected family members and was absent in unaffected adult members, available spouses and population controls.

Regarding the protein's domains, TMEM43 possesses sequences consistent with phosphorylation sites, a transactivation domain, YingOYang sites, a SUMO attachment site, an O-glycosylation site, and response element for PPAR gamma, although the functional significance of these domains in TMEM43 is still unknown. The p.S358L mutation occurs within the third of the protein's four trans-membrane spanning domains [2] and is predicted to disrupt the transmembrane helix according to Mutation Taster in silico analysis [10].

Although TMEM43 was depicted by Merner et al. [2] to be a cell membrane protein, studies in mouse neuroblastoma (N2a), Baby Hamster Kidney (BHK-21) and COS-7 cells show that TMEM43 localizes predominantly to the membranes of the nuclear envelope and endoplasmic reticulum [11]–[13]. Bengtsson and Otto found that TMEM43 is an ER protein enriched at the inner nuclear membrane [1]. They also showed that TMEM43 interacts with emerin as well as A- and B-type lamins. Similarly, a recent study also reported that TMEM43 may be a binding partner of LINC (linker of nucleoskeleton and cytoskeleton) associated with emerin and lamin of the nuclear envelope complex [14]. Fibroblast cells cultured from three patients with the p.S358L mutation demonstrate increased stiffness of the cell nucleus [5]. The TMEM43 protein can undergo homo-oligomerization which is dependent on transmembrane-spanning domain sequences [1], [15]. Despite the characterization of some of the possible TMEM43 binding partners, there have been limited studies on TMEM43 localization or the effects of the p.S358L mutation on the cardiac intercalated (IC) disc proteins. In COS-7 cells, the p.S358L mutation was not reported to result in a change in the solubility patterns of desmocollin-2, desmoglein-2, desmoplakin or junctional plakoglobin, although insoluble portions of desmocollin-2 and desmoplakin appeared to be reduced on immunoblots. No effects on lamin B or emerin were identified [13].

Most of the previously identified gene mutations that underlie ARVC (ARVC 8, 9, 10 and 12 and Naxos disease) are mutations of genes encoding desmosomal proteins: Plakophilin2 (PKP2), Desmoplakin (DSP), Desmoglein2 (DSG2), Desmocollin2 (DSC2) and Junction Plakoglobin (JUP) [16]–[23]. Oxford et al. have demonstrated that PKP2 silencing reduces the expression of the gap junction protein Cx43, particularly at the IC disc [24]. Saffitz and others have demonstrated in a mouse model that reduced electrical communication by gap junctions at the IC disc is associated with conduction slowing [25].

We and others have shown that PKP2 mutations reduce the expression of Cx43 gap junctions at the IC disc, which provides a possible explanation for the myocardial conduction delay that is typical of ARVC [26]–[29]. A PKP2 siRNA knock-down in cultured cardiomyocytes confirmed that the suppression of PKP2 also decreases sodium current and impairs cardiac action potential propagation [30]. This provides a possible explanation as to the delayed conduction and propensity to develop arrhythmias in ARVC. Others have shown that Cx43 expression is reduced early on in the disease, and that diminished expression of JUP is a sensitive and specific marker of autosomal dominant forms of ARVC. These expression changes are mainly due to mutations in desmosomal genes PKP2, DSP, DSG2 and DSC2 [31]. Whereas most forms of ARVC occur as a result of alterations to the proteins of the desmosome complex, which implicates these proteins in the disorder, the effect of TMEM43 mutations on IC disc protein expression has had limited study. We hypothesized that a TMEM43 mutation underlying ARVC5 causes changes in desmosomal or adherens junction proteins resulting in secondary gap junction changes at the IC disc.

Although the most serious consequences of ARVC are ventricular arrhythmias and sudden death, the atria are also involved in the disease [32], [33]. Thus, an HL-1 atrial cell line, which can maintain stable transfections in cell culture, was chosen to evaluate the TMEM43 p.S358L ARVC mutation. HL-1 cells (also frequently termed HL-1 cardiomyocytes) are an immortalized murine cell line derived from atrial tumour cells, which retain their adult cardiomyocyte contractile phenotype [34]. They are easily transfected with efficiencies of at least 75 to 80% [35]. HL-1 cells have been successfully used to investigate other arrhythmogenic cardiomyopathies including those due to RyR2 mutations (ARVC2) [36]–[42], mutations of the desmosomal gene desmocollin-2 (ARVC11) [43] and si-RNA studies of the desmosomal gene plakophillin-2 (ARVC9). We utilized methods for evaluation of HL-1 cell cultures which have been previously published, including protein expression [44] and immunofluorescence studies of cell-cell junction proteins [45]. Our group and others have also previously used microelectrode arrays (MEA) to look at conduction in cultures of HL-1 cells grown to confluence [46] [47].

We therefore sought to characterize the localization of TMEM43 in murine HL-1 cells, assess the effect of overexpression of wild-type or mutant TMEM43 on the expression patterns and localization of IC disc proteins and measure the resultant effects on electrical conduction and activation time as assessed by MEA.

## Results

### Expression and localization of TMEM43 in transfected HL-1 cells

**Figure 1:**
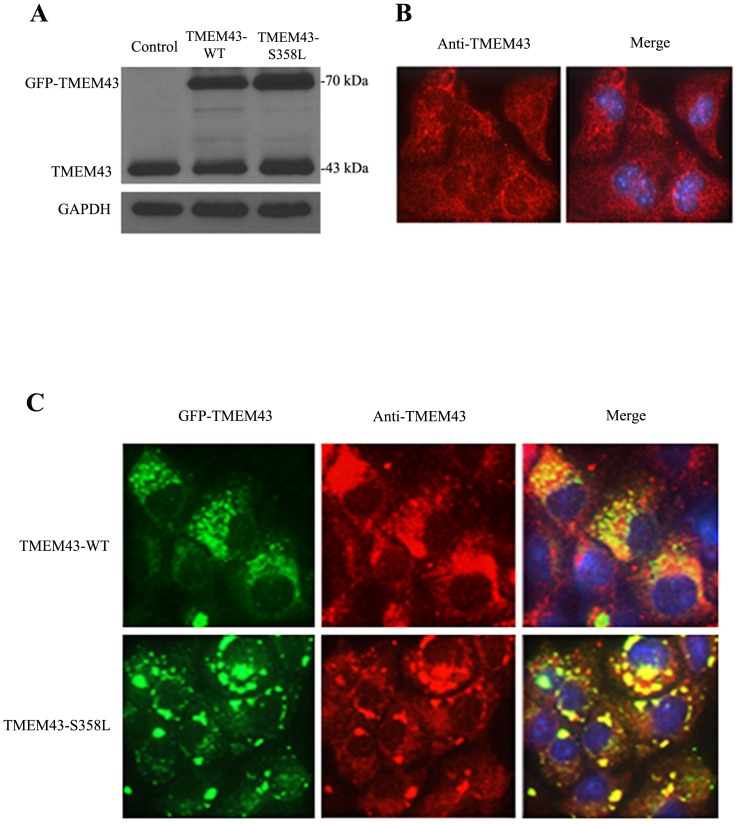
Expression and distribution of TMEM43 in non-transfected HL-1 cells and cells transfected with GFP-tagged wild-type or p.S358L TMEM43. **A**. Western blot analysis demonstrating expression of endogenous and exogenous (transfected) TMEM43, with GAPDH as loading control. Rabbit polyclonal TMEM43 antibody specifically detected GFP-TMEM43 and native TMEM43, indicated by the presence of bands at 70 kDa and 43 kDa respectively, in transfected cells and only 43 kDa native TMEM43 in non-transfected HL-1 cells (control). The 70 kDa band indicates the GFP-tagged wild-type (TMEM43-WT) or mutant (TMEM43-S358L) TMEM43 **B**. Immunofluoresence staining with TMEM43 antibody showed cytoplasmic as well as nuclear envelope distribution of endogenous TMEM43 in non-transfected HL-1 control cells. TMEM43 was detected using anti-TMEM43 antibody and Cy3-labeled anti-rabbit IgG. **C**. Immunofluorescence staining of TMEM43-WT or TMEM43-S358L cells. Expressed wild-type or mutant TMEM43 were observed by GFP fluorescence (left column); GFP-TMEM43 showed more cytoplasmic distribution. TMEM43 was detected in the cytoplasm of both the stably expressing wild-type (top row) and mutant (bottom row) cell lines. Nuclei are visualized with DAPI (blue, right column). GFP (green, left column) fluorescent, Cy3 (red, middle column) fluorescent and merged images (right column) are shown. Co-localization of signals due to GFP and antibody binding (yellow, right column).

Constructs consisting of full length wild-type and mutant (p.S358L) human TMEM43 tagged at the C-terminus with GFP were used to generate stably transfected HL-1 cells. Western Blot analysis with antibodies against TMEM43 highlighted the endogenous expression of murine TMEM43 at 43 kDa in non-transfected HL-1 cells. The presence of wild-type and mutant fusion proteins at approximately 70 kDa as well as similar endogenous TMEM43 expression at 43 kDa, were seen in the transfected cells (Figure 1A). The 43 kDa band remained unchanged despite the presence of either of the transfected TMEM43 proteins. The overexpression of either TMEM43 or the p.S358L mutant therefore does not affect the endogenous expression of TMEM43 in HL-1 cells. Immunofluorescence staining with TMEM43 antibody showed endogenous protein distribution in both cytoplasm and nuclear envelope in control cells (Figure 1B). GFP (green, Figure 1C left column) from the transfected fusion proteins co-localized (yellow-merge, Figure 1C right column) with antibody-stained TMEM43 (red, Figure 1C middle column). The TMEM43 antibody bound GFP-tagged TMEM43 and endogenous TMEM43 proteins in both the TMEM43-WT and TMEM43-S358L stable cell lines. The wild-type transfected protein (GFP-TMEM43-WT) was associated with more numerous and smaller circular puncta in cytoplasm. However, the mutant (GFP-TMEM43-S358L) protein showed reduced expression and altered morphology with decreased cytoplasmic distribution.

**Figure 2:**
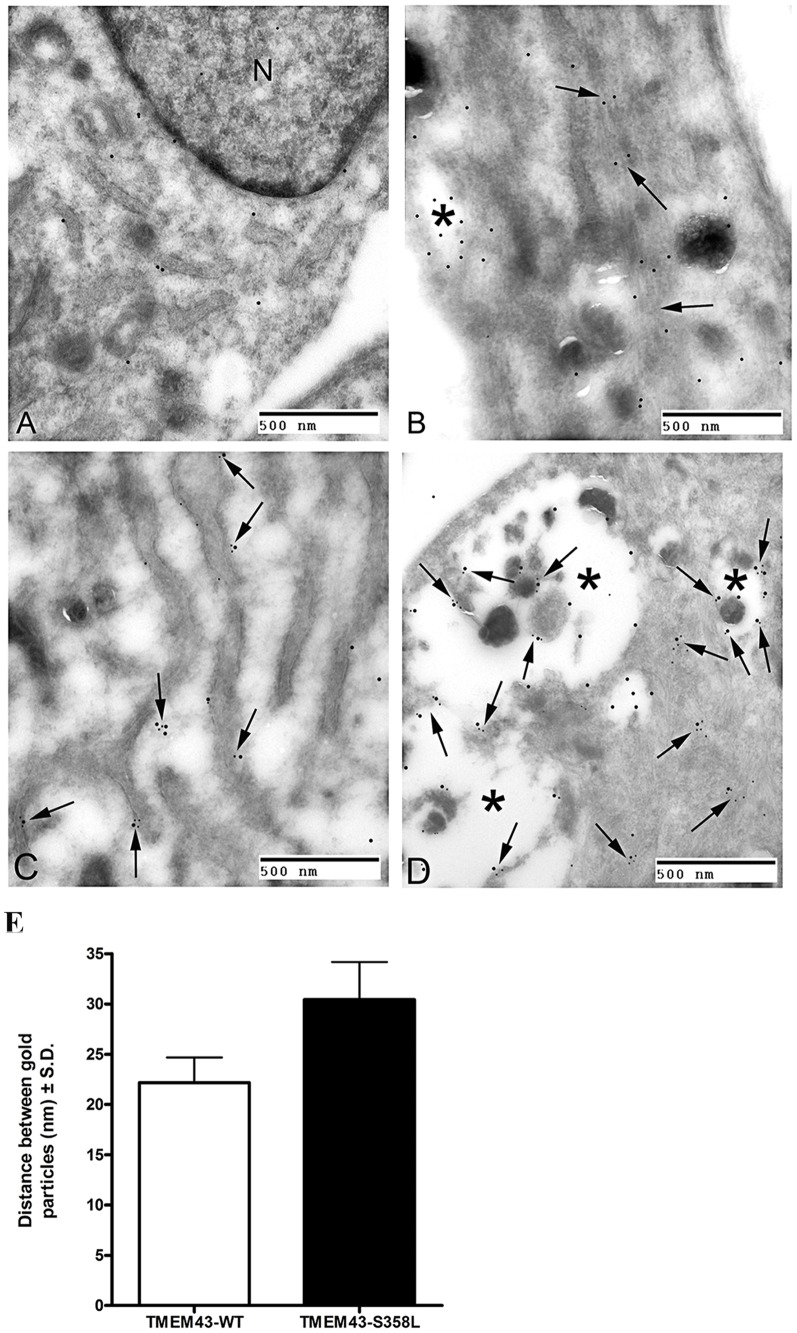
Transmission Electron Micrograph of transfected HL-1 cells labeled for TMEM43 with immunogold. **A** and **B**. Single immunogold labeling experiments used 15 nm gold particles to label GFP. **A**. Immunogold-labeled TMEM43-WT cells. The label (large dot) is associated with the endoplasmic reticulum (arrows), nuclear envelope, and the nucleus (N). **B**. Immunogold-labeled TMEM43-S358L cells. Immunogold particles (large dot) are confined to bundles of cytoplasmic microfilaments (arrows) and what vacuolated endosomal structures (asterisk). The endoplasmic reticulum or nuclear envelope did not show TMEM43 expression. **C** and **D**. Double immunogold labeling experiments used 15 nm gold particles to label GFP and 5 nm gold particles to label TMEM43. **C**. Cytoplasm of a wild-type cell that has been labeled with both TMEM43 (small dot) and GFP (large dot) antibodies. Note the co-localization on the endoplasmic reticulum (arrows). **D**. Mutant protein labeled with antibody specific for TMEM43 (small dot) or the GFP Tag (large dot) were found in a large vacuolated structure (asterisk) as well as discrete clusters in the cytoplasm (arrows) of stably transfected cells. All bars equal 0.5 µm. **E**. Quantitative analysis of the distance between large gold particles (GFP-15 nm) and small gold particles (TMEM43-5 nm) in TMEM43-WT (open column) and TMEM43-S358L (filled column) cells. Data are expressed mean ± SD from three independent experiments.

Electron microscopy (EM) of the perinuclear region of stably-transfected TMEM43-WT cells labeled for TMEM43 with immunogold demonstrate that the wild-type GFP-TMEM43 protein is predominately associated with the endoplasmic reticulum (Figure 2A and C). Immunogold labels for TMEM43 were also found on the nuclear envelope and often a sparse label was found within the nucleus (Figure 2A). In the TMEM43-S358L cells, labels were found primarily on bundles of cytoplasmic microfilaments as well as structures resembling phagosomes by appearance (Figure 2B and D). Immunogold labels against TMEM43 in TMEM43-S358L cells were rarely found on the endoplasmic reticulum, nuclear membrane or within the nucleus. It should be noted neither the endogenous nor GFP-TMEM43 protein were found in their standard distribution in the mutant cells. When double-labeling experiments were performed, the GFP (15 nm) was detected in a similar distribution to the TMEM43 (5 nm) (Figure 2C and D). In the TMEM43-WT cells (Figure 2C) the immunogold particles denoting GFP and TMEM43 are colocalized. The GFP antibody recognizes the GFP-TMEM43 protein and the TMEM43 antibody recognizes both the endogenous and GFP-TMEM43 protein. In TMEM43-S358L cells, the GFP is localized predominantly in phagosomes with some colocalization of the endogenous wild-type protein. Some of the fusion protein is preferentially localized to phagosomes compared to endogenous protein in the mutant cells (Figure 2D). This phagosomal distribution was not seen in the TMEM43-WT cells. In both TMEM43-WT and TMEM43-S358L cells there is some grouping of large and small particles in various ratios (3∶2, 1∶3, 1∶2) indicating TMEM43 homo-oligomerization, including between GFP-tagged and endogenous TMEM43. TMEM43-S358L protein appears to be pulling some endogenous TMEM43 into phagosomes, likely through homo-oligomerization, and this might explain the dominant negative effect of the S358L mutation causing a severe ARVC phenotype. No significant difference with respect to the average distance between the 15 nm gold particles (GFP) and 5 nm gold particles (TMEM43) was found in wild-type and mutant cells (Figure 2E).

### Abnormalities of IC disc proteins in HL-1 cells transfected with mutant TMEM43

**Figure 3:**
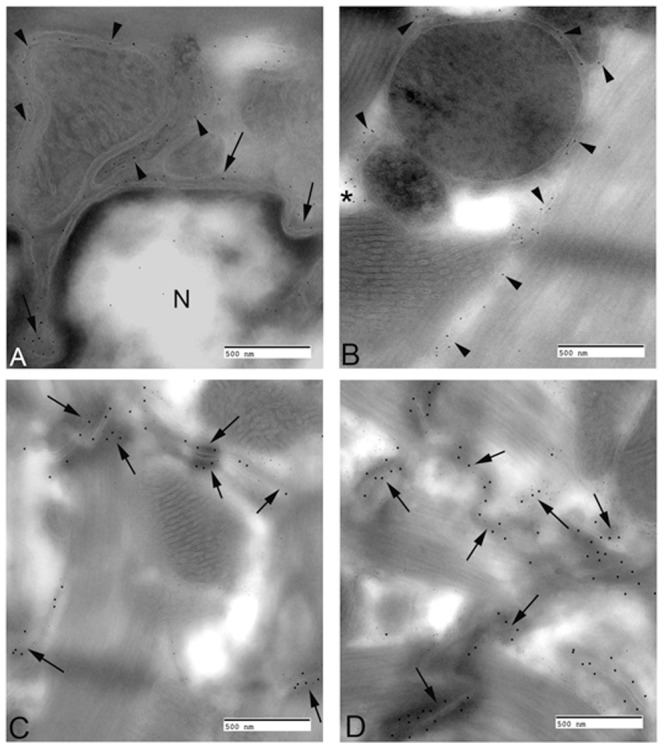
Expression of IC disc proteins in HL-1 cells transfected with wild-type and mutant TMEM43. **A** and **B**. The stably transfected cells were lysed and Western blot was performed for ZO-1, α-catenin, N-cadherin, β-catenin, JUP, Cx43 and GAPDH. The expression of α-catenin, N-cadherin, β-catenin and JUP were similar in non-transfected HL-1 (control) and stably-transfected cells (TMEM43-WT and TMEM43-S358L). **A**. The level of ZO-1 was significantly decreased in TMEM43-S358L cells compared to TMEM43-WT and control. **B**. P2 and P0 represent the phosphorylated and non-phosphorylated forms of Cx43. The level of the non-phosphorylated Cx43 isoform (P0) was increased in mutant cells compared to the control and TMEM43-WT cells. **C** and **D**. Densitometry analysis of ZO-1/GAPDH and phosphorylated Cx43 P2/P0. * denotes p<0.05. The densitometry results showed the combined analysis of three separate western blots and the western blot images are representative of three independent cell culture experiments.

To characterize the effect of the p.S358L mutation on IC disc proteins, Western blot analysis was performed on IC disc proteins from control, TMEM43-WT and TMEM43-S358L cells (Figure 3). When assessed by immunoblot, protein expression for α-catenin, N-cadherin, β-catenin and JUP, were not significantly altered (Figure 3A and B). There were, however, significant reductions in ZO-1 protein levels in the TMEM43-S358L cells (ZO-1/GAPDH ratio = 0.52), compared to TMEM43-WT cells (ZO-1/GAPDH ratio = 1.46, p<0.05) and control (ZO-1/GAPDH ratio = 1.55, p<0.05) (Figure 3C). There was no significant difference between the control and TMEM43-WT cells. Both the inactive non-phosphorylated (P0) and active phosphorylated (P2) forms of Cx43 were identified between ∼41 kDa and 43–45 kDa respectively on SDS-PAGE. The phosphorylation state has been previously shown to interfere with Cx43 migration in polyacrylamide gels resulting in decreased mobility and an inflated molecular weight [48]–[50]. The ratio of phosphorylated to non-phosphorylated Cx43 levels (P2/P0, Figure 3D) in TMEM43-S358L cells (Cx43 P2/P0 = 0.62) was significantly reduced compared to TMEM43-WT cells (Cx43 P2/P0 = 0.97, p<0.05. No significant difference was found between the control and TMEM43-WT cells.

**Figure 4:**
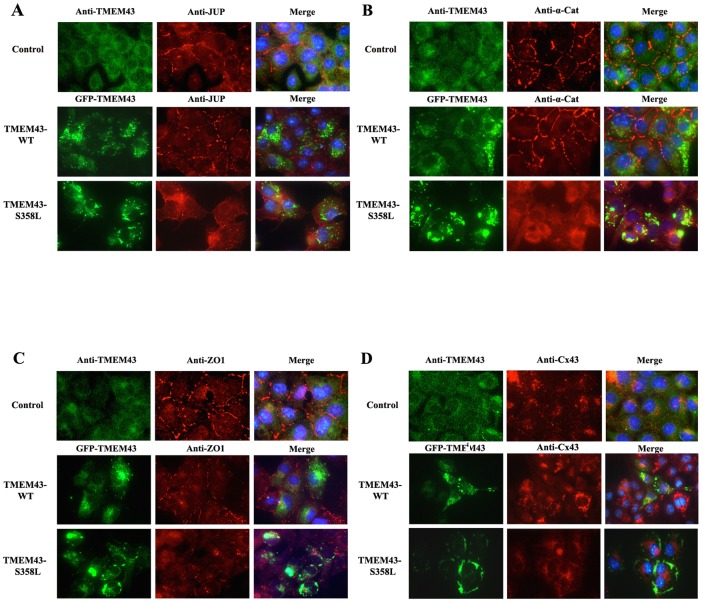
Immunofluorescent staining of TMEM43 and IC disc proteins in HL-1 cells stably expressing wild-type and mutant TMEM43. **A**. Immunostaining of Plakoglobin (JUP) was predominantly at the cell-cell border in control and TMEM43-overexpressing (TMEM43-WT) cells, but this significantly decreased at cell border in mutant TMEM43-transfected cells (TMEM43-S358L), with a diffuse expression throughout the cytoplasm instead. TMEM43 (green) was seen in the cytoplasm, as small spots in the TMEM43-WT cells. Greater cytoplasmic distribution was seen in control cells and near the cell edge in the TMEM43-S358L cells. **B**. α-catenin labeling clearly localized to the border of the cells in control and TMEM43-WT cells but in the TMEM43-S358L cells, α-catenin was clearly located diffusely throughout the cytoplasm, with very little staining specifically at the cell border. **C**. Immunostaining of ZO-1 labeling showed ZO-1 localization predominantly at the cell-cell junction in control and TMEM43-WT cells, while reduced amounts of ZO-1 were observed in TMEM43-S358L cells. **D**. Cx43 staining in TMEM43-WT cells demonstrates strong immunoreactive signals at the periphery of the cells and in the cytoplasm which is greater than those levels seen in control cells. In TMEM43-S358L cells, there is diffuse Cx43 staining of the cells throughout the cytoplasm. Images combining the TMEM43 staining/GFP fluorescence with these 4 different proteins (Merge) are shown in the right column including nuclear DAPI staining (blue). All results are representative of three independent cell culture experiments.

When stained by immunofluorescence, both control and TMEM43-WT cells show JUP, α-catenin and ZO-1 localization at cell-cell junctions (Figure 4A, B and C, middle column). However, there was a marked decrease of JUP, α-catenin, and ZO-1 staining at the cell-cell junction in TMEM43-S358L cells. JUP and α-catenin were clearly redistributed into the cytoplasm, with more α-catenin staining found in the cytoplasm of TMEM43-S358L cells when compared to TMEM43-WT and control (Figure 4A and B). ZO-1 expression was clearly diminished in the mutant cells (Figure 4C). β-catenin and N-cadherin staining were largely unchanged in control, TMEM43-WT and TMEM43-S358L cells, localizing predominantly to the cell border (Figure S1A and B). With respect to Cx43 immunofluoresence staining, TMEM43-WT cells showed defined labelling of structures predominantly at the periphery of the cells and greater cytoplasmic distribution when compared to control cells, whereas the staining in TMEM43-S358L cells was more diffuse throughout the cytoplasm (Figure 4D). We confirmed the interactions of wild-type TMEM43 in murine ventricular tissue sections, which clearly demonstrated the partial co-localization of TMEM43 (red) with JUP (green) on the cell membrane at the intercalated disc (arrows, Figure S2). Similarly the same co-localization pattern is observed with Cx43.

**Figure 5:**
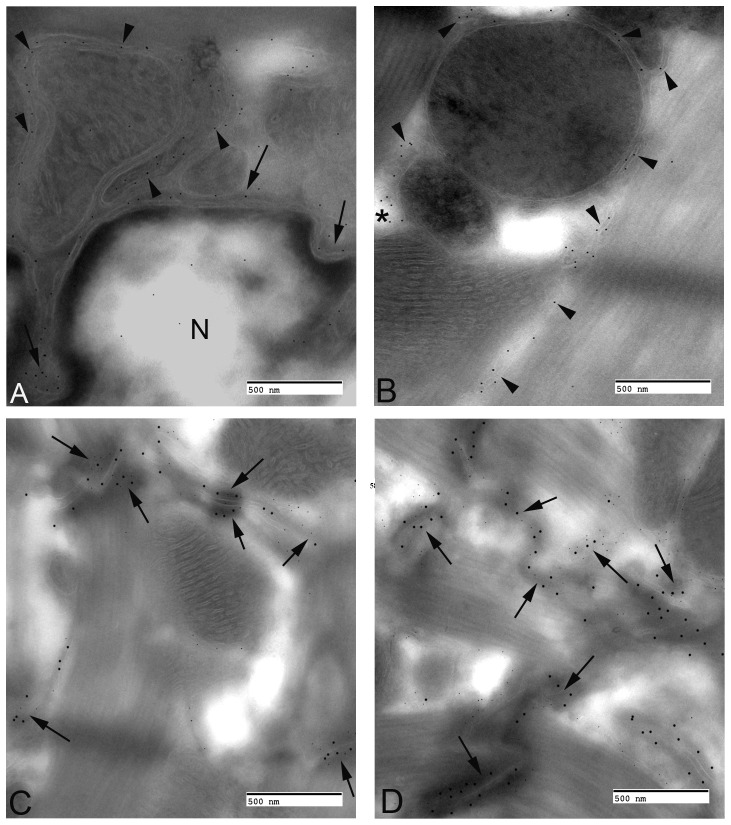
TMEM43 distribution and association with ZO-1 and JUP in the intercalated disc of normal murine myocardium. **A**. Electron micrograph of a double immunogold labeled section of the perinuclear region of a cardiac myocyte that has been labeled with TMEM43. Particles were located diffusely throughout the nucleus (N), on the nuclear membrane (arrows) and on components of the sarcotubular network (arrowheads). **B**. Sarcoplasm of a cardiac myocyte. Note the labeling on the sarcoplasmic reticulum (arrowheads) on vesicular components of the sarcotubular network (asterisk). Figure 4C represents co-localization of TMEM43 (10 nm gold particles) with ZO-1 (15 nm gold particles). Arrows indicate two adherens junctions that contain both ZO-1 and TMEM43. Figure 4D represents co-localization of TMEM43 (10 nm gold particles) with JUP (15 nm gold particles) in desmosomes and adherens junctions (arrows) of the intercalated disc. All bars equal 0.5 µm.

Our results confirm the findings of previous studies [11]–[13] showing that TMEM43 is predominantly found at the nuclear membrane and endoplasmic reticulum. However, its mutation appears to have major effects on the expression of adherens junction proteins such as ZO-1, α-catenin and plakoglobin at the intercalated disc. We therefore performed double immunogold labeling of normal murine myocardium to look for expression of TMEM43 protein in conjunction with these proteins. 5 shows an ultrathin cryosection of murine heart with TMEM43 colocalization with ZO-1 and other intercalated disc proteins. Figure 5A demonstrates the presence of TMEM43 on the nuclear membrane (arrows) and on components of the sarcoplasmic reticulum (Figure 5B, arrowheads). Figure 5C and D represents co-localization of TMEM43 with ZO-1 and JUP in desmosomes and adherens junctions (arrows) of the intercalated disc.

### Robotic Adherent Cell Injection

Gap junction permeability was evaluated using dye transfer experiments in confluent cardiomyocyte cultures. After incubation, the HPTS dye traveled from rhodamine-identified injected cells to the neighboring cells through functioning gap junction. The results in Figure S3 shows that the TMEM43-S358L group has less gap junctional intercellular communication (GJIC) than the TMEM43-WT and control group (1.60±0.21 cells vs. 3.42±0.40, p<0.001), and there is no significant difference (p>0.05) between the TMEM43-WT and control group. The results for the three groups are 1.60±0.21, 3.42±0.40, 4.57±0.36, respectively (mean ± standard error; student t-test p-values between 1&2, 2&3, 1&3, are<0.001, >0.05, <0.001, respectively).

### Reduced conduction velocity in HL-1 cells expressing mutated TMEM43

**Figure 6:**
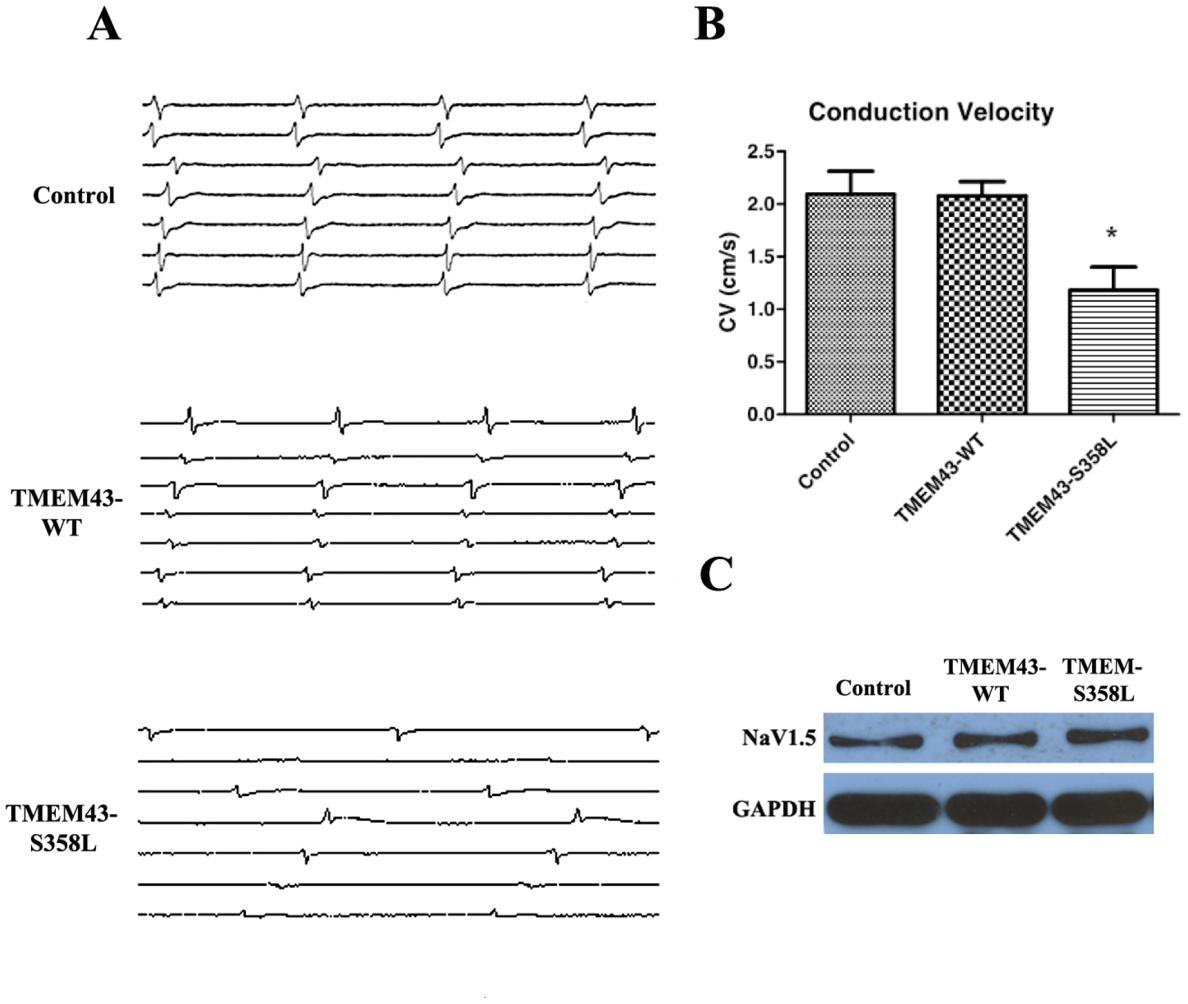
Effects of TMEM43 on conduction velocity and sodium channel expression. **A**. Electrograms recorded from adjacent MEA electrodes in HL-1 cell cultures. All cells beat spontaneously. Control and TMEM43-WT cells beat with a regular, synchronous rhythm. TMEM43-S358L cells beat with an irregular, comparatively slowed rhythm. **B**. Average velocity illustrating a decrease in conduction velocity of the TMEM43-S358L cells (1.2 cm/s) compared to control cells (2.5 cm/s) and TMEM43-WT cells (2.1 cm/s) (n = 7 control, n = 6 TMEM43-WT and n = 6 TMEM43-S358L, * p<0.01). All results are representative of six independent culture experiments. **C**. Western blot demonstrating expression of Na_V_1.5 in control, TMEM43-WT and TMEM43-S358L cells. GAPDH was used as a loading control. Rabbit polyclonal Na_V_1.5 antibody specifically detected sodium ion channel (Na_V_1.5) expression in all three cell types. Neither wild-type nor mutant TMEM43 altered the Na_V_1.5 expression. All results are representative of three independent Western blot experiments.

Recordings from confluent, stably-transfected cell cultures grown on microelectrode arrays allowed for assessment of both automaticity, defined as the intrinsic electrical rhythm of the HL-1 cells (either transfected or non-transfected), and electrical conduction. Examples of electrograms and conduction velocities recorded from control, TMEM43-WT and TMEM43-S358L cell cultures are shown in Figure 6A. Electrograms recorded the inherent, automatic rhythm of the plated cells by adjacent MEA electrodes showing that all cells beat spontaneously. The control and TMEM43-WT cells beat with regular, synchronous rhythms, whereas the TMEM43-S358L cells showed a slower, more irregular rhythm (Figure 6A). Figure 6B shows that averaged measured conduction velocity of TMEM43-S358L cultures (n = 6) was 1.2 cm/s, lower than both control (n = 7) and TMEM43-WT cultures (n = 6 each) at 2.5 and 2.1 cm/s respectively, illustrating a 40% reduction in conduction velocity (Figure 6B, p<0.01). (All cell cultures were paced at 2.5 Hz to exceed the fastest intrinsic automaticity) Our data suggest that the expression of the mutant TMEM43 in cells may disrupt the gap junction function, which is demonstrated by the failure to transfer small molecules and reduced cell-cell conduction. Activation maps during pacing for control, TMEM43-WT and TMEM43-S358L HL-1 confluent cardiomyocyte cultures are represented in Figure S4. Representative movies are also attached (Movie S1, S2, and S3). Western blot analysis demonstrated that expression of Na_V_1.5 (the main sodium channel expressed in heart) in control, TMEM43-WT and TMEM43-S358L were unchanged (Figure 6C). This finding suggests that the presence of TMEM43 did not alter overall sodium channel expression within cells.

## Discussion

Mutations in desmosomal proteins are by far the commonest genetic causes of ARVC among all implicated in the NCBI gene database (Table S1). TMEM43 is a recent gene mutation also implicated in ARVC. Unlike typical desmosomal gene mutations underlying ARVC, the function of TMEM43 and the mechanism by which its mutation causes disease are not well understood. Using the HL-1 (murine atrial cardiomyocyte) immortalized cell line as a model system, we evaluated the effect of a TMEM43 mutation on IC disc protein expression and localization as well as on conduction velocity. HL-1 cells are a well-established system and have been previously used to understand cardiac biology at the cellular and molecular levels [34], [35]. We generated HL-1 stable cell lines expressing either the C-terminal GFP-tagged wild-type TMEM43 (TMEM43-WT) or its GFP-tagged p.S358L mutation underlying ARVC5 (TMEM43-S358L). These stable cell lines were used to assess the effects of TMEM43 and the p.S358L mutation on IC disc protein expression and localization, as well as on conductance in confluent cardiomyocyte cultures. Although desmosomal gene mutations underlie most other forms ARVC, suggesting that this is a disease of the desmosome, the gene mutation in TMEM43 causing ARVC5 primarily affects proteins associated with adherens and gap junctions.

### Location of TMEM43

TMEM43, also called LUMA (nuclear membrane protein), has been localized predominantly to the inner nuclear membrane in multiple non-cardiac cell types and also shows some expression outside the nucleus, including the endoplasmic reticulum [1]. TMEM43 is thought to have a role in maintaining the nuclear envelope structure, interacting with lamins A and B and retaining emerin at the inner nuclear membrane [14]. A recent report in COS-7 cells expressing TMEM43 demonstrates that the protein localizes to the nuclear envelope. Its S358L mutation has no effect on nuclear envelope proteins [13], but does appear to increase stiffness of the cell nucleus based on studies of fibroblast from patients with this mutation [5]. TMEM43 has been shown to localize to the endoplasmic reticulum and inner nuclear envelope (mouse neuroblastoma cells, COS-7, BHK, HeLa and other cell lines) and the GFP-tagged protein has also been shown to localize to the ER/inner nuclear envelope sites [1], [12], [13]. Our data show that both endogenous and exogenous tagged TMEM43 can be detected in the cytoplasm and the nuclear envelope regions suggesting that the tag does not affect the TMEM43 localization. Our studies using immunogold electron microscopy of transfected cultured HL-1 cells further confirm the location of TMEM43 at the nuclear envelope and endoplasmic reticulum. Our studies of normal murine myocardium demonstrate that TMEM43 does colocalize with ZO-1 and JUP proteins at the adherens junction of the IC disc. This is in keeping with TMEM43 being a component of adherens junctions in epithelia and composite junctions of myocardial IC discs (colocalizing with β-catenin) [51]. It is not currently known how TMEM43 interacts with IC disc proteins but it is becoming increasingly recognized that the IC disc is a dynamic structure [52]. Both electrical and mechanical functions of the IC disc components seem to be dependent on microtubular trafficking of vesicles to and from the plasma membrane [53]. Our transmission electron microscopy and immunogold staining showed that the over-expressed wild-type TMEM43 localized to the nuclear envelope and endoplasmic reticulum (Figure 2A and C). When the p.S358L mutant was overexpressed, there was an increased localization of the mutant protein in the vacuolated structures (Figure 2B and D). This suggests that the highly conserved serine residue on the third membrane spanning region of TMEM43 may have a role in stabilizing the protein in the membrane or trafficking the protein to its appropriate cellular compartment. The mutant protein may be recognized as misfolded and selected for degradation, which may account for the increased incidence of the mutant of protein in vacuolated structures resembling phagosomes.

### Effects of p.S358L mutation on IC disc proteins

Little is known about TMEM43 function beyond it containing a response element for PPAR gamma, an adipogenic transcription factor [2]. We have demonstrated that the presence of p.S358L affects the expression and distribution of IC disc proteins ZO-1, α-catenin, JUP, as well as Cx43 (Figure 4). The most interesting findings were that the p.S358L mutation resulted in lower expression levels of ZO-1, abnormal localization of JUP and α-catenin, and altered phosphorylation of Cx43. N-cadherin and β-catenin are less involved in the conduction defects in TMEM43-S358L cells as their expression and localization at the IC disc is preserved (Figure S1). One study has demonstrated with immunohistochemistry that ARVC patients with mutations in the TMEM43 gene have a lower expression of JUP at the IC disc in the myocardium [4] compared to a normal control. Our study demonstrates that localization of JUP at the cell-cell junction was altered significantly with JUP being clearly dispersed into the cytoplasm in mutant TMEM43-S358L cells as seen by immunofluoresence (Figure 4A). However, we found no significant expression difference of JUP in TMEM43-S358L cells compared with TMEM43-WT by Western blot analysis. This result is in agreement with a previous study showing more cytoplasmic distribution in the hearts of two patients with the TMEM43 mutation [4].

### TMEM43 effects on ZO-1 and connexin-43 phosphorylation

TMEM43 may be implicated in phosphorylation of Cx43, either directly or through alteration of levels of ZO-1. ZO-1 is involved in signal transduction at cell-cell junctions [54], binds to catenins and is closely associated with both catenins and Cx43 during gap junction assembly [55], [56]. Recently it was shown that the interaction of ZO-1 and Cx43 is important for novel mechanisms which regulate membrane permeability and intercellular communication, which the authors hypothesize would lead to conduction disturbances [57]. Changes of ZO-1 may therefore lead to the redistribution of α-catenin and JUP to the cytoplasm [58]–[60], a result similar to seen when the proteins are coexpressed with p.S358L.

Altered localization of α-catenin and JUP may also have secondary effects on the modification of Cx43. Changes in ZO-1 has been shown to decrease the Cx43 P2/PO ratio via reductions in the phosphorylated isoform (P2) [58]–[60]. Interestingly, the expression of the non-phosphorylated form (P0) of Cx43 was increased in cells expressing the p.S358L mutant relative to the phosphorylated form (P2). Furthermore, the relative amount of Cx43 (both forms) seemed to be elevated whenever TMEM43 or the p.S358L mutant was overexpressed. It has been reported that gap junction assembly is associated with phosphorylation to the P2 form and that the P0 form is predominantly intracellular [61]. These findings are consistent with our experiments such that the P2 form of Cx43 demonstrated in TMEM43-WT cells was the predominant state. However, the most abundant form in the TMEM43-S358L cells was the P0 Cx43. Phosphorylation of Cx43 is important in regulating assembly, stability and function of gap junctions [49], [62]–[65]. We speculate that the phosphorylation of Cx43 may be involved in the proper trafficking of Cx43 from the ER to the membrane. Interestingly the p.S358L mutation in TMEM43 leads to altered Cx43 phosphorylation in stably-transfected HL-1 cells adding credence to the hypothesis that the protein may be involved in locally regulating the gap junction complex.

Gap junction function appears to be established when Cx43 interacts with ZO-1 and is impaired when there is interference with that interaction. Changes in the Cx43 phosphorylation state is one possible mechanism for slowing cell-to-cell conduction and causing the reduced conduction velocity observed with TMEM43-S358L cells. This could be responsible for the resultant arrhythmias seen in ARVC type 5. Initial studies from Sato *et al.* provided the first evidence of the association of Plakophilin-2 (PKP2) with sodium channels in impaired conduction propagation [30]. However, in the presence of p.S358L, the Nav1.5 sodium channel expression appears to be unaffected. The effect of TMEM43 on sodium channels remains to be explored.

## Conclusions

The TMEM43 missense mutation (p.S358L) found in ARVC type 5 patients affects localization of IC disc proteins and conduction velocity through unknown mechanisms. Our results show that one possible mechanism of disturbed conduction in TMEM43-S358L cells involves localization defects of α-catenin and JUP, a down-regulation of ZO-1 expression and changes in phosphorylation state of the gap junction protein Cx43. These events may modify proteins in adherens and gap junctions leading to decreased conduction velocity. The pathophysiology by which TMEM43 mutation leads to altered cardiac IC disc proteins, conduction delay and ventricular arrhythmias of ARVC is still poorly understood and needs to be further investigated. By increasing the understanding of ARVC pathology, specific therapies can be developed to prevent the arrhythmias and deaths associated with this disease. Since TMEM43 seems to have a role in locally regulating gap junctions, this may become a useful pharmacological target.

## Methods

### Generation of cDNA constructs

To evaluate the pathogenic potential of the TMEM43 mutations, we cloned the wild-type human TMEM43 and TMEM43 missense mutation (p.S358L) into expression vectors [2]. PCR was performed using the clone TMEM43-IRAU13-C10 containing full length human TMEM43, forward primer 5′- GATGCTAGCATGGCCGCGAATTATTCCAG-3′ and reverse primer 5′-GCAGAATTCGCTCCAACTTTTTGGCTGGCAC-3′. The resulting PCR products were directionally cloned by EcoRI and NheI sites in pEGFP-N1 expression vector (Clontech, Mountain View, CA, USA) to generate plasmid p-h-TMEM43, which contains cDNA tagged with green fluorescent protein (GFP) at the C-terminus. Mutated p-h-mTMEM43 was obtained by site directed mutagenesis of the wild-type construct by using the QuickChange Site-Directed Mutagenesis Kit (Stratagene, La Jolla, CA, USA). The following mutagenic primers were used: 5′-CTGTGTGGCCACCTTGCTGACCCTGCT-3′ and antisense 5′-AGCAGGGTCAGCAAGGTGGCCACACAG-3′. The plasmids were verified by sequence analysis.

### HL-1 cell line culture and transfection with wild-type and mutant TMEM43

HL-1 cells were received from Dr. William C. Claycomb [34] (Louisiana State University Health Science Center, New Orleans, LA, USA). The cells were grown in Claycomb medium (JRH Biosciences, Lenexa, KS, USA) supplemented with 10% fetal bovine serum (JRH Biosciences, Lenexa, KS, USA), 0.1 M norepinephrine (Sigma, Saint Louis, Missouri, USA), 2 mM L-Glutamine (Invitrogen, South San Francisco, CA, USA), and penicillin/streptomycin (10^4^ U/ml P and 10^4^ µg/ml S; Invitrogen, South San Francisco, CA, USA) in a humidified 5% CO_2_ incubator at 37°C. HL-1 cells were plated on fibronectin-coated plates as monolayers on glass coverslips at a density of approximately 2×10^5^ cells/well in a 6-well plate, and as monolayers on MEA plates at a density of 6×10^6^ cells/plate. HL-1 cells were transfected with the p-h-TMEM43 and p-h-mTMEM43 plasmids using FuGENE HD Transfection Reagent (Roche, Indianapolis, IN, USA) according to the manufacturer's instructions to create stable cell lines (TMEM43-WT and TMEM43-S358L). After 48 hrs, fresh growth media was supplemented with 0.5 mg/ml G418 (Invitrogen, South San Francisco, CA, USA) as the selection reagent. The selection was allowed to proceed for 21 days, and the selected colonies were harvested and maintained in the selection media. The HL-1 cell lines will be referred to as follows: non-transfected HL-1 cells (control), stably-transfected GFP-tagged wild-type TMEM43 cell line (TMEM43-WT), and stably-transfected GFP-tagged mutant TMEM43 cell line (TMEM43-S358L).

### Primary antibodies

Rabbit polyclonal anti-TMEM43 (N-13) antibody (Santa Cruz, CA, USA) was used in immunofluorescence microscopy and immunoblot analysis to detect TMEM43. We used mouse monoclonal antibodies of anti-α-catenin, anti-ß-catenin, anti-ZO-1, anti-N-cadherin, anti-JUP, and anti-Cx43 (Invitrogen, San Francisco, CA, USA), rabbit anti-Nav1.5 antibody (Abcam, MA, USA) and mouse anti-GAPDH (SIGMA, Saint Louis, Missouri, USA) for detection of these proteins in immunoassays.

### Immunogold labelling of cultured HL-1 cells and murine myocardium

Stably-transfected cells were fixed in 4% paraformaldehyde containing 0.1% glutaraldehyde in 0.1M phosphate buffer for 2–4 hours and stored in PBS containing 20 mM Na Azide at 4°C. Prior to cryoultramicrotomy, cells were rinsed thoroughly in phosphate buffer and infused with 2.3M sucrose overnight. The cells were then mounted on cryoultramicrotomy pins and frozen in liquid nitrogen. Ultrathin cryosections were then cut in a cryoultramicrotome at −120°C and transferred in a loop of molten sucrose to formvar coated nickel grids. Sections of both TMEM43-WT and TMEM43-S358L lines were then washed in PBS containing 0.15% glycine and 0.5% BSA. Following rinses in PBS/BSA the grids were incubated with a rabbit polyclonal TMEM43 antibody for 1 hour, rinsed in PBS/BSA and incubated with a goat anti-rabbit IgG gold complex for an additional hour. Sections were then washed with PBS and distilled water before being stabilized in a thin film of methylcellulose containing 0.2% uranyl acetate. They were then examined and photographed using a transmission electron microscope (TEM) (JEOL JEM-1011). Measurements were conducted on a minimum of ten fields per culture from three different cultures per group. In double labeling experiments, the cultured cells were not stabilized in methyl cellulose until the end of the labeling protocol. Following TMEM43 labeling, the samples were washed thoroughly in PBS/BSA and incubated in a murine monoclonal antibody against GFP for an additional hour. Again the samples were washed thoroughly followed by incubation in a 15 nm gold goat anti-mouse gold complex. The grids were then stabilized in a thin film of methylcellulose containing uranyl acetate and examined and photographed in the TEM. Controls included the omission of the primary antibody in single labeling experiments or either the first or second primary antibody in double labeling experiments.

For immunogold experiments, the hearts of 6-week old mice were fixed in 4% paraformaldehyde in 0.1 M Phosphate buffer PH 7.2 containing 0.1% glutaraldehyde and processed for ultrathin cryomicrotomy. For antigen labeling, ultrathin cryosections were labeled separately with antibodies specific for TMEM43 (rabbit polyclonal anti-TMEM43 [N-13] antibody, Santa Cruz) or either ZO-1 (mouse monoclonal anti-ZO-1 antibody, Zymed), or Junction Plakoglobin (JUP) in the myocardium (mouse monoclonal anti-JUP antibody, Zymed). Secondary labeling of TMEM43 was performed with 10-nm gold-labeled fragments from goat anti-murine IgG. Specimens were observed through a JEOL JEM-1011 transmission electron microscope.

### Immunofluorescence of cultured HL-1 cells and murine myocardium

Control, TMEM43-WT and TMEM43-S358L cells were plated on glass coverslips, washed in PBS, and fixed in cold methanol for 10 min at −20°C. The cells were then rinsed with PBS and blocking buffer (PBS with 2% donkey serum, 1% bovine serum albumin, 0.1% Triton X-100 and 0.05% Tween 20) and then blocked for 1 hr. The cells were then incubated with mouse monoclonal primary antibodies, anti-α-catenin, anti-ZO-1, anti-Cx43, anti-ß-catenin, or anti-N-cadherin for 1 hr. After three rounds of rinsing with PBS, the corresponding Cy3-conjugated donkey anti-mouse IgG (Jackson ImmunoResearch, West Grove, PA) was added for another 30 min at room temperature.

For double staining experiments, after the secondary antibody was applied, the cells were washed, permeabilized, and blocked with a rabbit anti-TMEM43 polyclonal antibody. The cells were then washed and treated with a Dylight-488-conjugated donkey anti-rabbit secondary antibody. DAPI staining was performed and, after a final rinsing step, sections were mounted with a 10% solution of polyvinyl alcohol containing 2.5% 1,4-diazabicyclo-2,2,2-octane (PVA/DABCO, Sigma, St. Louis, MO, USA). Cells were visualized using a Nikon Eclipse E1000 microscope attached to a cooled CCD camera (QImaging, Retiga EX) and a computer-generated video analysis system (Image-Pro Plus software, Media Cybernetics, Silver Springs, MD).

For mouse immunofluorescence experiments, 5 µm fresh hearts slides were fixed in cold acetone for 15 min at −20°C, followed by rinsing with PBS and blocked in PBS with 2% normal donkey serum, 1% bovine serum albumin (BSA), 0.1% Triton X-100 and 0.05% Tween-20 for 1 hour at room temperature, and incubated with the mouse anti-JUP (1∶100, Zymed Laboratories), rabbit anti-Cx43 (1∶200, Zymed Laboratories) antibodies for 1 hour. After three rounds of rinsing with PBS, the corresponding cy3-conjugated donkey anti-mouse or anti-rabbit IgG (1∶500 dilution, Jackson ImmunoResearch, West Grove, PA) were added for another 30 min at room temperature. Finally DAPI staining was performed to identify nuclei. After a final rinsing step, sections were mounted with a 10% solution of polyvinyl alcohol containing 2.5% 1,4-diazabicyclo-2,2,2-octane (PVA/DABCO, both from Sigma, St. Louis, MO, USA), coverslipped, and visualized using a confocal microscope (60× magnification).

### Western Blot Analysis

Cells were rinsed twice with PBS, sonicated for 15 sec in 50 µl of RIPA lysis buffer (20 mM Tris-HCl, pH 8.0; 1% Nonidet P-40, 0.1% SDS, 0.5% sodium deoxycholate, with complete protease inhibitor; Roche), and centrifuged at 12,000 rpm for 15 min. Protein concentration was determined with the Bradford protein assay (Bio-Rad, Hercules, CA, USA). Aliquots of 50 µg of protein extracts were separated on 10% SDS-polyacrylamide gel electrophoresis (SDS-PAGE) and transferred to nitrocellulose membranes. Membranes were blocked with 5% milk in Tris-buffered saline (TBS), cut and separately probed with rabbit anti-TMEM43, mouse anti-Cx43, anti-ZO-1, anti-N-cadherin, anti-α-catenin, anti-ß-catenin, anti-JUP, or anti-GAPDH for 2 hrs. After washing (TBS/Tween 0.05%), the HRP-conjugated secondary antibodies, goat anti-mouse (Santa Cruz Biotechnology Inc.) or goat anti-rabbit IgG-HRP (Santa Cruz Biotechnology Inc.), were incubated for 1 hr followed by wash and detection of chemiluminescence by ECL reagents (Santa Cruz, Biotechnology, USA). For quantification of ZO-1 and Cx43 in the cultured transfected cells, blots for ZO-1, Cx43 (P2/P0), and GAPDH (loading control) were run in triplicate. Densitometric analysis was performed with Quantity One (Bio-Rad). The ratio of Cx43 phosphorylation (Cx43 P2/P0) and ZO-1 expression (ZO-1/GAPDH) in TMEM43-S358L cultures were compared to TMEM43-WT and control cells. Ratios were determined for Cx43 phosphorylation states (P2/P0) and for ZO-1 compared to the GAPDH loading controls (ZO-1/GAPDH). These ratios were compared between TMEM43-S358L, TMEM43-WT, and control cell lines.

### Robotic Adherent Cell Injection

Dye transfer of a small molecule dye from a microinjected cardiomyocyte is an accepted method of assessing cardiomyocyte cell-cell coupling at connexin-43 gap junctions [66]. A small molecule fluorescent dye, 8-Hydroxypyrene-1, 3, 6-trisulfonic acid, trisodium salt (HPTS) (Sigma, St. Louis, MO, USA) similar to Lucifer Yellow (molecular weight 524 v. 457) was used for dye transfer assessment in our experiments. HL-1 cardiomyocytes were maintained in Claycomb medium supplemented with 10% FCS, P/S (100 µgml^−1^), epinephrine (0.1 mM) and L-glutamine (2 mM) as previously described [28]. We adapted an automated robotic cell injection system to inject HPTS to quantitatively measure the gap junctional intercellular communication (GJIC) in confluent control, TMEM43-WT and TMEM43-S358L transfected HL-1 cells. This method measures the number of neighboring cells taking up HPTS dye after a 15 minute incubation period. More than 30 injections per cell culture plate were performed.

### Microelectrode Array Conduction Recording

Unipolar electrograms were recorded using a standard microelectrode array (MEA) (200/10iR-Ti-gr, MultiChannel Systems, Reutlingen, Germany) consisting of a planar 60-channel gold MEA organized in a 8×8 grid with no electrode at the corners, designed with a diameter of 20 µm and an electrode-to-electrode pitch of 200 µm. The MEA plates were cleaned and coated with gelatine/fibronectin (Sigma, St. Louis, MO, USA). The control, TMEM43-WT and TMEM43-S358L cells beat spontaneously once confluence has been reached (2–4 days in culture) at which time conduction measurements were performed. Each MEA was connected to a 60-channel amplifier (MultiChannel System) for recording extracellular potentials. A separate platinum reference electrode was immersed in the solution away from the recording electrodes. Temperature was controlled and set at 37°C. Electrograms were digitized and recorded using a proprietary data acquisition and analysis system (isochrone and velocity measurement) developed using custom Matlab software. Areas of automaticity within the culture were identified, and conduction velocity of wavefronts emanating from these areas was calculated. To compensate for differences in automaticity between samples, conduction velocity was also measured during stimulation of the confluent HL-1 cultures using a paired wire electrode connected to Grass Instrument S88X (Grass Instruments, Warwick RI). Stimulus voltage was adjusted to a threshold to achieve local stimulation without field stimulation, at a stimulus frequency of 2.5 Hz. This frequency was chosen to ensure 1∶1 capture on all HL-1 culture types and stimulation was applied for 30 s prior to recording. Temperature was controlled and set at 37°C. For optical mapping, the cardiomyocyte monolayers were loaded with 5 µM di-4-ANEPPS dye (Invitrogen, Burlington, Ontario, Canada) for 5 min in the dark at 37°C, and washed 3 times in Tyrode salt solution. A complementary metal oxide semiconductor (CMOS) camera system (Micam Ultima –L, Brainvision, USA) was used to acquire the optical data as previously described [46]. Optical signals were monitored during a 30 s period to ensure 1∶1 capture and local stimulation. Waves radiating away from the pacing site with no wavebreak were identified for measuring conduction velocity.

### Animals and ethical approval

The current study was approved by the Animal Care Committee (ACC) at the Toronto Centre for Phenogenomics (TCP Animal Use Protocol: 0155), Toronto, Canada. C57BL/6 mice (6 wks of age, Jackson Laboratory) were housed and cared for at the TCP animal facility according to ethical guidelines and regulations.

### Statistical Analysis

All data are presented as mean +/− standard deviation. To determine statistical significance, the non-parametric test, Mann-Whitney U test was performed to compare the data obtained from wild type to mutant transfected cells. For comparison between 3 groups, one-way analysis variance was performed. A p-value of less than 0.05 was considered significant.

### Study Limitations

TMEM43 was tagged with GFP at the C-terminus in our study. The C-terminus of TMEM43 is not a known binding domain for any known protein, but the functional domains of TMEM43 are not well described and it is still unclear which functional domains are actually involved in protein-protein interactions. We minimized the impact of this limitation by comparing to endogenous TMEM43 in both the transfected and non-transfected cells. Assessment of TMEM43 location, its protein interactions and the effect of the p.S358L mutation mutation on other IC disc proteins in normal and ARVC5 disease patient tissue would be ideal, but such tissues are rarely available, and rarely collected using methods that allow for these studies. Another study limitation includes the sample size used. Our results are based on the analysis conducted using five samples each from both TMEM43-WT and TMEM43-S358L cell lines.

## Supporting Information

Figure S1 **Immunofluorescence staining of IC disc proteins β-catenin and N-cadherin in HL-1 cells expressing wild-type or mutant TMEM43.** **A**. β-catenin staining is predominantly in the edges of cell-cell contact and there was little change between control, TMEM43-WT and TMEM43-S358L cells. **B**. Immunostaining of N-cadherin is confined to the cell-cell contact sites, with little change between control, TMEM43-WT and TMEM43-S358L cells. Images combining the TMEM43 staining with β-catenin and N-cadherin proteins (Merge) are shown in the right column including nuclear DAPI staining (blue). All results are representative of three independent cell culture experiments.
(TIF)

Figure S2 **Immunofluorescence staining of IC disc proteins junctional plakoglobin (JUP) and Cx43 in mouse myocardium.** **A**. Mouse sections clearly demonstrated the partial co-localization of TMEM43 (red) with JUP (green) on the cell membrane at the intercalated disc (arrows). **B**. Similarly the same co-localization pattern is observed with Cx43. Images combining the TMEM43 staining with JUP and Cx43 proteins (Merge) are shown in the right column including nuclear DAPI staining (blue).
(TIF)

Figure S3 **Assessment of cell-cell molecule transport in control, TMEM43-WT and TMEM43-S358L transfected HL-1 cells using HTPS/rhodamine dye.** Using a robotic microinjection system, HPTS dye (8-Hydroxypyrene-1, 3, 6-trisulfonic acid, trisodium salt) were injected in confluent control, TMEM43-WT and TMEM43-S358L transfected HL-1 cells. The HPTS dye after incubation, traveled from rhodamine-identified incised cells to the neighboring cells through functioning gap junction. The number of adjoining cells uptaking the fluorescent dye from the injected cells was counted as a measure to investigate the gap junction function. The results are expressed as mean ± Standard error for three groups control (4.57±0.36), TMEM43-WT (3.42±0.40) and TMEM43-S358L (1.60±0.21) transfected cells. p<0.001 (control vs TMEM43-WT and TMEM43-S358L), p>0.05 (control vs TMEM43-WT) respectively.
(TIF)

Figure S4 **Effects of TMEM43 on Activation Maps during pacing.** The monolayer preparations were electrically stimulated at 2.5 Hz with a bipolar electrode located on the right side of each map. All maps have a normalized scale of 400 ms (1 cycle). **A**. Activation map from a control HL-1 monolayer cell culture. The map shows rapid conduction radiating from the pacing electrode. **B**. Activation map from a TMEM43-WT monolayer cell culture with an activation spread similar to the previous panel. **C**. Activation map from a TMEM43-S358L monolayer cell culture. Slower activation spread can be seen.
(TIF)

Table S1 **Published ARVC mutations.**
(PDF)

Movie S1 **Illustrative example showing fast activation of a HL-1 control monolayer preparation during 2.5 pacing.**
(AVI)

Movie S2 **TMEM43-WT preparation shows a similar propagation speed as observed in control.**
(AVI)

Movie S3 **A significant slowing of activation propagation can be seen in mutant TMEM43-S358L, along with wave breaks.**
(AVI)
