# Supplementary figures and images for: TMEM43 Mutation p.S358L Alters Intercalated Disc Protein Expression and Reduces Conduction Velocity in Arrhythmogenic Right Ventricular Cardiomyopathy
Source: PLoS One. 2014 Oct 24;9(10):e109128. doi: 10.1371/journal.pone.0109128 (PMC4208740; doi:10.1371/journal.pone.0109128)

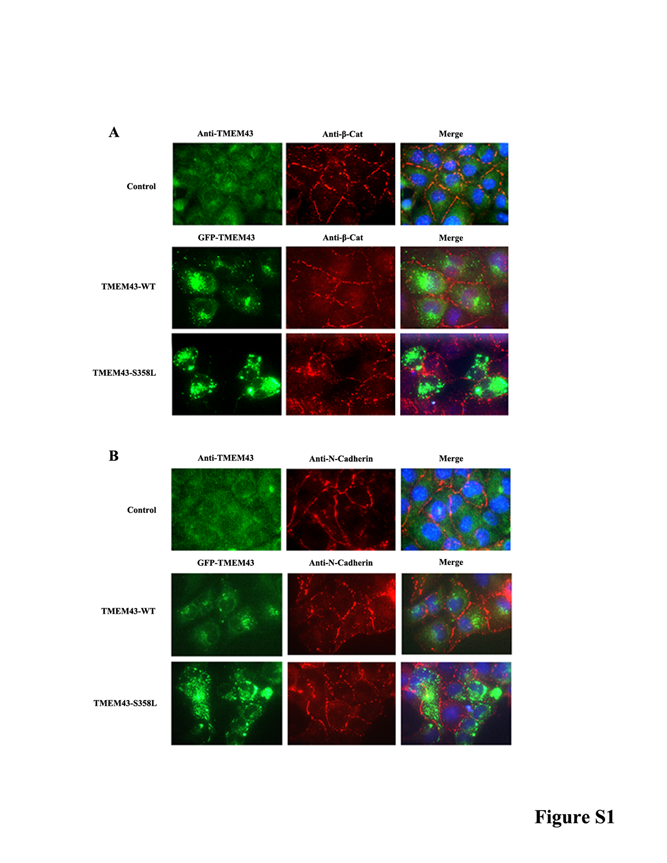

Supplement: Figure S1 — Immunofluorescence staining of IC disc proteins β-catenin and N-cadherin in HL-1 cells expressing wild-type or mutant TMEM43. A. β-catenin staining is predominantly in the edges of cell-cell contact and there was little change between control, TMEM43-WT and TMEM43-S358L cells. B. Immunostaining of N-cadherin is confined to the cell-cell contact sites, with little change between control, TMEM43-WT and TMEM43-S358L cells. Images combining the TMEM43 staining with β-catenin and N-cadherin proteins (Merge) are shown in the right column including nuclear DAPI staining (blue). All results are representative of three independent cell culture experiments. (TIF) [file pone.0109128.s001.tif]

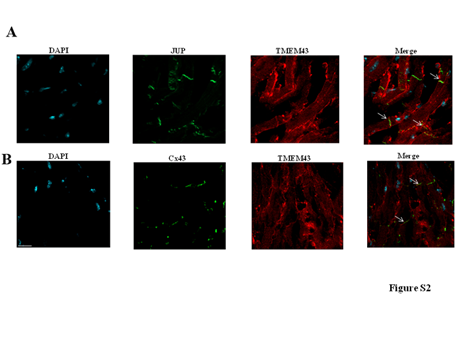

Supplement: Figure S2 — Immunofluorescence staining of IC disc proteins junctional plakoglobin (JUP) and Cx43 in mouse myocardium. A. Mouse sections clearly demonstrated the partial co-localization of TMEM43 (red) with JUP (green) on the cell membrane at the intercalated disc (arrows). B. Similarly the same co-localization pattern is observed with Cx43. Images combining the TMEM43 staining with JUP and Cx43 proteins (Merge) are shown in the right column including nuclear DAPI staining (blue). (TIF) [file pone.0109128.s002.tif]

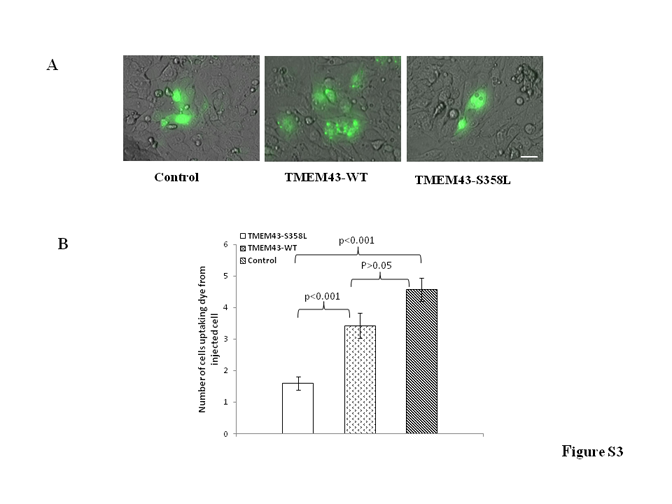

Supplement: Figure S3 — Assessment of cell-cell molecule transport in control, TMEM43-WT and TMEM43-S358L transfected HL-1 cells using HTPS/rhodamine dye. Using a robotic microinjection system, HPTS dye (8-Hydroxypyrene-1, 3, 6-trisulfonic acid, trisodium salt) were injected in confluent control, TMEM43-WT and TMEM43-S358L transfected HL-1 cells. The HPTS dye after incubation, traveled from rhodamine-identified incised cells to the neighboring cells through functioning gap junction. The number of adjoining cells uptaking the fluorescent dye from the injected cells was counted as a measure to investigate the gap junction function. The results are expressed as mean ± Standard error for three groups control (4.57±0.36), TMEM43-WT (3.42±0.40) and TMEM43-S358L (1.60±0.21) transfected cells. p<0.001 (control vs TMEM43-WT and TMEM43-S358L), p>0.05 (control vs TMEM43-WT) respectively. (TIF) [file pone.0109128.s003.tif]

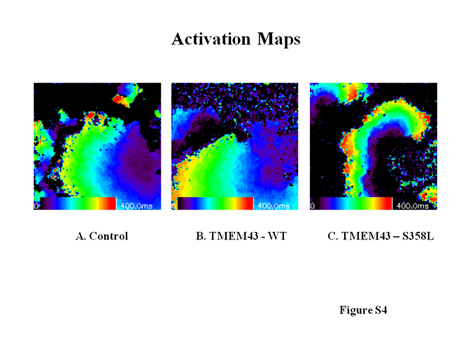

Supplement: Figure S4 — Effects of TMEM43 on Activation Maps during pacing. The monolayer preparations were electrically stimulated at 2.5 Hz with a bipolar electrode located on the right side of each map. All maps have a normalized scale of 400 ms (1 cycle). A. Activation map from a control HL-1 monolayer cell culture. The map shows rapid conduction radiating from the pacing electrode. B. Activation map from a TMEM43-WT monolayer cell culture with an activation spread similar to the previous panel. C. Activation map from a TMEM43-S358L monolayer cell culture. Slower activation spread can be seen. (TIF) [file pone.0109128.s004.tif]
