# Supplementary material for: TMEM43 Mutation p.S358L Alters Intercalated Disc Protein Expression and Reduces Conduction Velocity in Arrhythmogenic Right Ventricular Cardiomyopathy
Source: PLoS One. 2014 Oct 24;9(10):e109128. doi: 10.1371/journal.pone.0109128 (PMC4208740; doi:10.1371/journal.pone.0109128)
Supplement: Table S1 — Published ARVC mutations. (PDF) [file pone.0109128.s005.pdf]

Table S1. Published ARVC mutations[1]

Type	Gene	Locus	Function
ARVC1	TGFβ3	14q23-q24	Transcription factor ligand, modulates cell adhesion and promotes myocardial fibrosis [2]
ARVC2	RYR2	1q42-q43	Calcium channel for calcium-induced calcium release (CICR), induces the release of calcium from the sarcoplasmic reticulum into the cytosol [3]
ARVC3	Unknown	14q12-q22	Unknown
ARVC4	Unknown	2q32.1-q32.3	Unknown
ARVC5	TMEM43	3p25	Unknown
ARVC6	Unknown	10p14-p12	Unknown
ARVC7	Unknown	10q22.3	Unknown
ARVC8	DSP	6p24	Anchors intermediate filaments to desmosomal plaques [4]
ARVC9	PKP2	12p11	Role in transcriptional regulation and links cadherens to intermediate filaments in desmosome plaque [5]
ARVC10	DSG2	18q12.1-q12	Calcium-binding transmembrane glycoprotein, involved in the interaction of plaque proteins and mediates cell-cell adhesion [6]
ARVC11	DSC2	18q12.1	Calcium-dependent glycoprotein member of the cadherin superfamily may contribute to desmosome and gap junction interdependence through connexin43 regulation [7]
ARVC12	JUP	17q21	Common junctional plaque protein. Forms complexes with cadherins and desmosomal cadherins (DSC2 and DSG2), plays a central role in the structure and function of submembranous plaques [8]

The table reflects the current available information of the ARVC mutations. Data was obtained from NCBI gene data base[1].

References

1. McNally E, MacCleod H, Dellafave L (2005) Arrythmogenic Right Ventricular Dysplasia/Cardiomyopathy, Autosomal Dominant. NCBI Gene. http://www.ncbi nlm.nih.gov/books/NBK1131/
2. Beffagna G, Occhi G, Nava A, Vitiello L, Ditadi A, Basso C, Bauce B, Carraro G, Thiene G, Towbin JA, Danieli GA, Rampazzo A (2005) Regulatory mutations in transforming growth factor-beta3 gene cause arrhythmogenic right ventricular cardiomyopathy type 1. Cardiovascular research 6: 366–73.
3. Jiang D, Wang R, Xiao B, Kong H, Hunt DJ, et al (2005) Enhanced store overload-induced Ca2+ release and channel sensitivity to luminal Ca2+ activation are common defects of RyR2 mutations linked to ventricular tachycardia and sudden death. Circulation research 97: 1173-1181.
4. Rampazzo A, Nava A, Malacrida S, Beffagna G, Bauce B, Rossi V, et al (2002) Mutation in human desmoplakin domain binding to plakoglobin causes a dominant form of arrhythmogenic right ventricular cardiomyopathy. Am. J. Hum. Genet 71: 1200-1206.
5. Mertens C, Hofmann I, Wang Z, Teichmann M, Sepehri CS, Schnolzer M, Franke WW (2001) Nuclear particles containing RNA polymerase III complexes associated with the junctiona plaque protein plakophilin 2. Proc. Nat. Acad. Sci. 98: 7795-7800.
6. Nekrasova OE, Amargo EV, Smith WO, Chen J, Kreitzer GE, Green KJ (2011) Desmosomal cadherins utilize distinct kinesins for assembly into desmosomes. J Cell Biol. 195: 185-203.
7. Gehmlich K, Lambiase PD, Asimaki A, Ciaccio EJ, Ehler E, Syrris P, Saffitz JE, McKenna WJ (2011) A novel desmocollin-2 mutation reveals insights into the molecular link between desmosomes and gap junctions. Heart Rhythm. 8: 711-8.
8. Delmar M, McKenna WJ (2010) The cardiac desmosome and arrhythmogenic cardiomyopathies: from gene to disease. Circulation research. 107: 700-14.
